# Mycobacterium marinum infections in Denmark from 2004 to 2017: A retrospective study of incidence, patient characteristics, treatment regimens and outcome

**DOI:** 10.1038/s41598-018-24702-7

**Published:** 2018-04-30

**Authors:** Inge K. Holden, Michala Kehrer, Aase B. Andersen, Christian Wejse, Erik Svensson, Isik Somuncu Johansen

**Affiliations:** 1 Department of Infectious Diseases, Odense University Hospital, Odense, Denmark; 2 Department of Infectious Diseases, Rigshospitalet, Copenhagen, Denmark; 3 Department of Infectious Diseases, Aarhus University Hospital, Aarhus, Denmark; 4 International Reference Laboratory of Mycobacteriology, Statens Serum Institut, Copenhagen, Denmark

## Abstract

*Mycobacterium marinum* (*M. marinum*) is a slowly growing nontuberculous mycobacterium. The incidence of *M. marinum* infections in Denmark is unknown. We conducted a retrospective nationwide study including all culture confirmed cases of *M. marinum* from 2004 to 2017 in Denmark. All available medical records were reviewed. Demographics, clinical characteristics, and treatment regiments were analyzed. Fifty-five patients were identified, 40 (72.7%) were men with a median age of 50 years. Aquatic exposure was reported by 48 (90.6%) of the patients. Site of infection was upper extremities in 49 (92.5%) patients and 49 (92.5%) had superficial infection. The median time from symptom presentation to diagnosis was 194 days. All patients received antibiotics. Median time of treatment duration among all patients was 112 days. Treatment outcome was classified as improved in 40 (75%), improved with sequela in 4 (7.6%) patients and only 3 patients (3.8%) were classified as failed. Infection with *M. marinum* is rare and there is a long delay from symptom manifestation to diagnosis. The infection is predominantly related to aquatic exposure. *M. marinum* should be a differential diagnose in patients with slow-developing cutaneous elements and relevant exposure. Treatment outcomes are overall good and severe sequela are rare.

## Introduction

*Mycobacterium marinum (M. marinum)* is a slowly growing nontuberculous mycobacterium, which was first isolated from marine fish*. M. marinum* is found in non-disinfected salt- and freshwater reservoirs such as swimming pools and fish tanks^1,2^. The bacteria may cause infections in humans when contaminated water or infected fish or seafood come in contact with skin lesions^3^.

Most infections with *M. marinum* are superficial skin infections characterized by granuloma and lymphangitis also known as “fish tank granuloma” or “swimming pool granuloma”; however, the infection may spread to deeper tissue causing tendinitis, arthritis and osteomyelitis^3^.

The incidence of *M. marinum* infections in Denmark is unknown. In France in 1996–1998, the incidence was reported to be 0.04 per 100 000 person years^4^. Recent studies from The United States reported an increase in incidence of cutaneous nontuberculous mycobacterial infections and *M. marinum* infections in particular^1,5^.

The treatment of *M. marinum* infection is long-term antibiotic treatment and in the more severe cases surgery may be needed^1,5,6^. The optimal duration of antibiotic treatment is not known; as little as four weeks of antibiotic treatment has been reported as successful but often the treatment duration will be extended to several months^6–15^.

This study was conducted to provide information regarding the incidence, clinical features, treatment regimens and clinical outcome of patients infected with *M. marinum* infections in Denmark.

## Methods

We conducted a nationwide retrospective study in Denmark from January 1^st^, 2004 to May 31^st^, 2017.

All patients with a positive culture of *M. marinum* were included. Patients were followed from the time of registration of microbiological material at International Reference Laboratory of Mycobacteriology until the end of treatment, lost to follow-up, or May 31^st^, 2017, whichever came first.

The International Reference Laboratory of Mycobacteriology (IRLM) at Statens Serum Institut (SSI) is the only laboratory which performs all cultures, culture-based drug susceptibility testing, and molecular typing of *M. tuberculosis* complex and nontuberculous mycobacteria in Denmark^16^.

Cultures were performed in Mycobacterium Growth Indicator Tube system (MGIT; Becton Dickinson, Sparks, MD) and on Loewenstein- Jensen (LJ) incubated at 35 °C. For skin specimens and specimens for which *M. marinum* culture was requested, a supplemental LJ tube was inoculated and incubated in 33 °C. *M. marinum* was identified with nucleic acid techniques varying over the study period. Microscopy for acid-fast bacilli (AFB) using auramine-rhodamine stain were performed on all specimens.

We retrieved data from the laboratory register at IRLM on patients with positive *M. marinum* culture in the study period. *M. marinum* cases were cross-linked with administrative and medical registers using the unique Danish civil registration number, which is assigned to all residents of Denmark at birth or after residing legally in Denmark for 3 months. Medical records were reviewed for the following variables: age, sex, immunocompromised status (HIV, organ transplantation, chronic corticosteroid use or other immunosuppressive drug use, or other immunocompromised condition (i.e. inflammatory bowel disease, rheumatoid arthritis)), history of aquatic exposure and type, localization and severity of infection (cutaneous or subcutaneous infections, ulcerations, noduli, lymphangitis, tenosynovitis, arthritis, and osteomyelitis), time of onset of symptoms, diagnostic methods, any radiographic findings, perioperative findings, antibiotic treatment (type, duration, and cause of treatment change as well as having received empiric antibiotics prior to diagnosis), outcome and date of last registered contact.

The time to diagnosis was defined as the time from onset of symptoms to diagnosis of *M. marinum* infection by microscopy or culture.

Infection severity was classified into four clinical categories^17,18^: localized superficial cutaneous lesions (type I), nodular lymphangitis or with nodules, abscesses and granulomas (type II), deeper infections with or without skin involvement (type III), and disseminated infection (type IV).

In accordance with previous definitions, four treatment outcomes were defined: (a) “improved” if no recurrence of disease after cessation of antibiotic treatment, (b) “improved with morbidity” if sign of improvement but without return of full function of the affected limb, (c) “failed” if there was recurrence of disease after cessation of antibiotic treatment or persistence of disease despite ongoing therapy at time of last contact, and (d) “lost to follow-up” if no documentation of outcome or treatment in medical records^7^.

The initial antibiotic treatment regimen was defined as the first treatment regimen that was prescribed, while backbone antibiotic regimen was defined as the regimen used for the majority of the treatment period^7^.

Surgery was defined as having any surgery besides biopsy or aspiration.

### Ethics

This study was approved by the Danish Data Protection Agency (Jnr. 16/13130 and 2012-54-0100) and the Danish Health Authority (Jnr 3-3013-1640/1). In accordance with Danish law, observational studies performed in Denmark do not need approval from the Medical Ethics Committee or written consent from participants. All analyses are presented anonymously.

### Analyses

All cases were included in the incidence rate analyses, but only cases with available medical records were used in the following analyses.

Continuous variables were described using medians with interquartile range (IQR), and categorical variables were described using frequency counts and percentages.

When appropriate, differences between categories were analyzed using the t test for qualitative values. Comparisons were made using the Student’s t-test, and a p-value of <0.05 was considered statistically significant.

The yearly population of Denmark on July 1 came from Statistics Denmark^19^. The population was defined as contributing to 1 person year per resident per year in the incidence rate analyses. The crude annual incidence rates were calculated as the number of incident cases per 100 000 person years with the corresponding 95% confidence intervals (95% CI).

The statistical analyses were performed using Stata® (StataCorp., College Station, TX, USA, version 15.0).

## Results

From January 1^st,^ 2004 to May 31^st,^ 2017, we identified 55 patients with culture verified *M. marinum* infections. No medical records were available for 2 patients, both treated in non-hospital settings.

**Figure 1:**
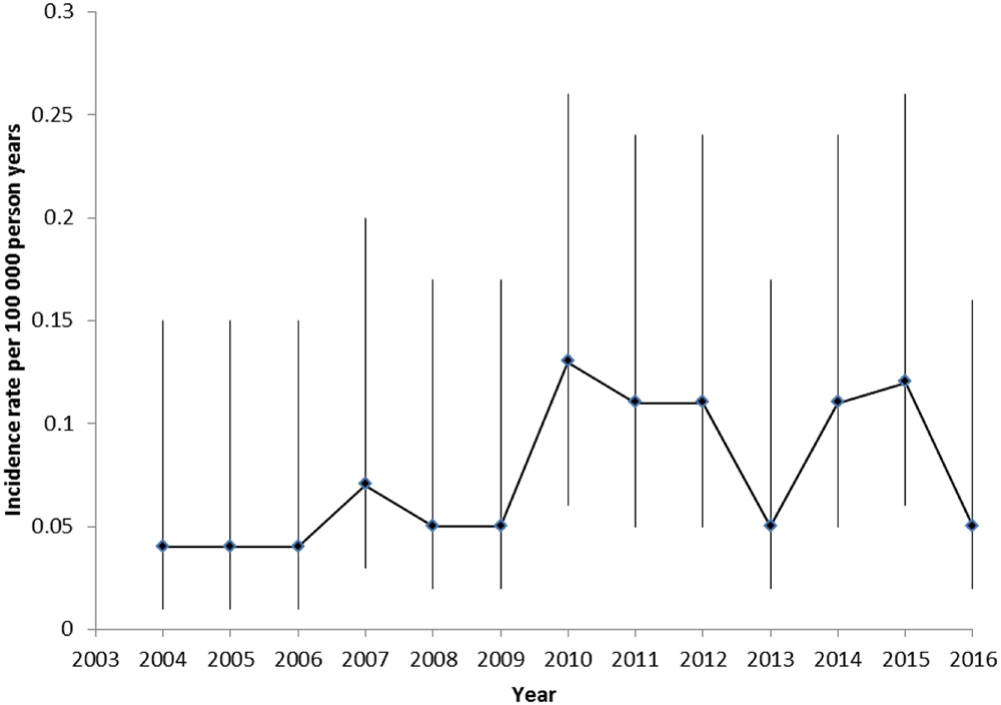
Incidence rate per 100 000 person years of *M. marinum* infection in Denmark with 95% confidence intervals. The population was defined as contributing to 1 person year per resident per year in the incidence rate analyses.

The incidence rates are shown in Fig. 1. The incidence rate during 2004 to 2009 was 0.04–0.06 per 100 000 person-years, from 2010 to 2016 the incidence rate was 0.05–0.13 per 100 000 person-years.

In total, 38 (71.7%) of the patients were men and the median age was 49 years (IQR: 36–59). The general patient characteristics, comorbidity and clinical findings are listed in Table 1. Almost all patients had a history of aquatic exposure (90.6%), the most common exposure came from maintenance of fish tanks. For the remaining 5 patients, the exposure was unknown. Type I infection was the most frequent (66%) and finger/hand (88.7%) was the most common site of infection.

**Table 1:** Characteristics and diagnostic methods used in patients with *M. marinum* infection in Denmark.

Variables	Number (%)
Total	53^a^
Men	38 (71.7)
Age in years: median	49 (IQR: 36–59)
Immunocompromised	9 (17.0)
**History of aquatic exposure:**	
Total	48 (90.6)
Fish tank	44 (83.0)^b^
Fishing	3 (5.7)
Handled fish/seafood	2 (3.8)
Swimming/diving	1 (1.9)
**Severity of infection:**	
Type I	35 (66.0)
Type II	14 (26.4)
Type III	2 (3.8)
Type IV	2 (3.8)
**Localization of infection:**	
fFinger/hand	47 (88.7)
iInvolved arm	17 (32.1)^c^
lLeg	3 (5.7)
Involved joints	2 (3.8)^d^
**Diagnostic methods:**	
Microbiological:	
Acid-fast bacilli stain positive	7 (13.2)
Culture from biopsy or aspiration or material from surgical procedures	53 (100)
**Pathology examination:**	
Granulomatous inflammation	35 (66.0)^e^
No granulomatous inflammation or positive acid-fast bacilli stain	4 (7.6)
**Imaging performed:**	
X-ray only	5 (9.4)
MRI	3 (5.7)^f^
PET-CT	3 (5.7)^g^
None	43 (81.1)

^a^Two patients with missing medical records were left out of the analyses.

^b^Two patients reported exposure to fish tank and fishing.

^c^Fifteen patients had involvement of finger/hand and arm.

^d^One patient had involvement of joint and finger/hand and arm.

^e^Forty-two patients had histological examination performed.

^f^Three patients had X-ray and MRI performed.

^g^Three patients had X-ray and PET-CT performed, one patient had X-ray, MRI and PET-CT performed.

The median time from symptom presentation to diagnosis was 194 days (IQR: 80–548). A total of 21 patients received empiric antibiotic therapy, 9 patients received a regimen which was effective against *M. marinum* infection, 6 patient received beta-lactam antibiotics, and 1 patient received dapsone. For the remaining patients, information regarding the empiric antibiotic regimen was not accessible.

The incubation period could not be assessed since time of initial infection was unknown for the majority of the patients.

### Treatment regimens and duration

The treatment regimens and median duration are listed in Table 2.

**Table 2:** Treatment of patients with *M. marinum* infection in Denmark.

Total	Number (%)
	53
**Backbone antibiotic regimen** ^a,b^	
One drug	25 (48.1)
Two drugs	18 (34.6)
Three drugs	8 (15.4)
Four drugs	1 (1.9)
**Treatment duration in days, median (IQR):**	
Overall:	112 (64–203)
Type I	120 (83–143)
Type II	74 (30–180)
Type III	284 (284–284)
Type IV	145 (50–240)
Regimens including:	
Rifampicin and ethambutol	176 (112–284)
Clarithromycin and ethambutol	123 (87–244)
Rifampicin, clarithromycin and ethambutol	176 (112–244)
**Outcome:**	
Improved	40 (75.5)
Improved with morbidity	4 (7.5)
Failed	3 (5.7)
Lost to follow up	6 (11.3)
Outcome: “improved” for one drug regimen (n = 27) including:	
Tetracyclines (n = 16)^c^	14 (87.5)^d^
Macrolides (n = 6)^e^	6 (100.0)
Outcome: “improved” for 2–4 drug regimen (n = 27) including^f^	
Rifampicin and ethambutol (n = 11)	7 (63.6)
Clarithromycin and ethambutol (n = 15)	9 (60.0)
Rifampicin, clarithromycin and ethambutol (n = 9)	5 (55.6)
NOT including clarithromycin and ethambutol (n = 12)	9 (75.0)

^a^The antibiotic regimen used for the majority of the treatment period.

^b^One patient received multiple combinations of antibiotics; backbone therapy could not be assessed.

^c^Ten patients received doxycycline and 6 patients received tetracycline.

^d^Two patients were lost to follow up.

^e^Five patients received clarithromycin and one patient received azithromycin.

^f^Clarithromycin was the only macrolide used in the 2–4 drug regimens.

All patients were treated with antibiotics either as monotherapy (45.3%) or as a combination of up to four antibiotics. Only 6 patients (11.3%) needed surgery performed.

All patients were treated by specialists in dermatology, infectious diseases, or pulmonary diseases. Only 11 patients (20%) were treated by dermatologists in non-hospital settings; 10 of these patients had type I infection and one patient had type II infection. All patients treated in non-hospital settings received a one-drug regimen, and median treatment duration was 80 days (IQR: 42–113). The most frequent drug of choice was doxycycline (36.4%) and median treatment duration was 91 days (IQR: 61–126). Outcome was classified as improved for 10 patients and 1 patient was classified as failed.

The majority of patients treated in hospital settings was diagnosed with type I infection (61.9%). Of those with type I infection, 10 patients (38.5%) received a one-drug treatment. Among all patients treated in hospital settings, 18 (43.9%) received a two-drug backbone regime and the overall median treatment duration was 123 days (IQR 84–244). The most commonly used combination in the 2, 3, and 4 drug backbone regimes were rifampicin and clarithromycin (38.9%).

Overall superficial infection (type I and type II) accounted for 92.5% of the cases, with a median treatment time of 110 days (IQR: 64–183). Fifty-one percent received a one drug regime and doxycycline was the most common drug prescribed (40%). The median treatment duration was 87 days (IQR: 42–130).

Among patients with deep tissue infection (type III and type IV), median time of treatment was 240 days (IQR: 50–284). They all received a treatment regime of at least 2 drugs, clarithromycin and ethambutol were included in all these treatment regimes.

### Treatment outcome

The majority of the patients (75.5%) were classified as improved at treatment termination. Four patients were classified as improved with morbidity, two of these patients had type IV infection, and one patient had a finger amputated. The other patient received immunosuppressive therapy and had extensive scar tissue related to the sites of infection. The last two patients had type I infection; one of these patients was diagnosed with inflammatory bowel disease and received immunosuppressive therapy. At treatment termination, both patients were described having moderate scar tissue related to the site of infection.

Only 3 patients (5.7%) were classified as failed. In this group, two patients were diagnosed with rheumatoid arthritis and received immunosuppressive therapy at time of diagnosis. The first patient was treated for a type III infection for more than 5 years, treatment was discontinued several times, but the patient quickly showed signs of relapse, the patient was still receiving treatment as the study was concluded. The second patient was diagnosed with type I infection and was treated for 897 days but had signs of relapse at follow-up. The last patient had type II infection, was not immunocompromised, received a one drug regimen for 15 days and at the last visit the lesions were described as slightly improved and still visible.

## Discussion

In this nationwide study, we reported 55 cases of culture confirmed *M. marinum* infections during a 13-year period. Infections with *M. marinum* are rare and related to aquatic exposure in Denmark. There is a long delay in diagnosis, but treatment outcome is overall good. However, severe manifestation of infection may be correlated with long treatment duration and need of surgery and sequelae.

More than 90% of the patients reported aquatic exposure. This is in consensus with the fact that *M. marinum* has been isolated from marine animals, fresh and saltwater^2^. The most common risk factor identified was fish tank maintenance, yet the incidence of *M. marinum* infections among owners of fish tanks is unknown. The last survey in Denmark regarding pet owners was completed in 2000 and reported that 80.000 families own fish tanks^20^ this is less than 2% of families in Denmark. This indicates that the incidence of *M. marinum* infection among fish tank owners in Denmark is very low. However, there has been reported an increase in number of families (10%) owning fish tanks in other countries^2^, which has been correlated to an increase in incidence of *M. marinum* infections during the last decade^1^. Yet, we were not able to identify a significant increase in the incidence rate of *M. marinum* infections in Denmark.

One patient in our study reported swimming pool as the sole known exposure. Before chlorination of swimming pools, several outbreaks of *M. marinum* infections caused by swimming in pools were reported in the literature^21,22^. Today, this is an unusual mode of transmission. Outbreaks of *M. marinum* have been reported in fish farms^8^ and fish markets during the last decade^6^. In our study, there were no clustered reports of *M. marinum* cases, hence no suspicion of an outbreak.

The majority of the patients in this study reported location of infection corresponding to upper extremities (92.5%). This correlates with the most common exposure: maintenance of fish tanks, hence finger/hands are the most common exposed areas. Several previous studies have likewise reported upper extremities as the most common infection site^1,8,10–14,23,24^.

Median time from symptom onset to diagnosis in this patient group was 194 days, earlier studies have reported great variance in delay of diagnosis (median 35–105 days)^1,6,24^. Both patient delay and doctor’s delay contributed to the delay of diagnosis. The majority of patients had had symptoms for weeks to months before consulting a doctor. The doctor’s delay could be attributed to the rare nature of *M. marinum* infection and the fact that cutaneous elements can resemble a staphylococcus infection. *M.marinum* will typically be detected with a mycobacterial culture performed at a lower temperature than usual. Unless the necessary information was lacking, all specimens from skin or from patients who had a suspected *M. marinum* infection were also cultured at a lower temperature. However, a delay in diagnosis was not significantly related to a poorer outcome in this study.

Our study consisted of patients with culture confirmed *M. marinum* infection, but only 13.2% had a positive AFB microscopy. Previous studies have also reported a low occurrence of positive AFB microscopy among patients with *M. marinum* infection (0–39%)^6,8,11,12,14,24,25^, indicating that a negative AFB microscopy cannot rule out the diagnosis of *M. marinum* infection and must be accompanied by culture.

Only 19% of patients had imagining performed. However more than 90% of our patients had type I or type II infection, which is compatible with superficial cutaneous infection. Therefore, in the majority of these cases there will not be an indication for imaging. Previous studies have shown a similar distribution of the severity of infection, hence *M. marinum* infection is more commonly a superficial infection that is limited to the skin. Most deep tissue infections with *M. marinum* have been reported as case studies in older and or immunocompromised patients^1,18,26–28^. This correlates with our results that only 4 patients (8%) had deep tissue infection and 2 of these patients received immunosuppressive therapy.

The results of this study revealed that 24 (45%) of the patients received a one-drug regimen. All of these patients had superficial cutaneous infection (type I: 80%, type II: 20%). Reported outcome was good; 88% of these patients were classified as improved and only one patient was classified as treatment failure at last contact. The remaining two patients in the group treated with a one-drug regimen were lost to follow-up.

Infections with *M. marinum* are rare, consequently there have not been any trials comparing treatment regimens and the optimal combination of antibiotics and treatment duration remains unknown. In Denmark there is no national guideline regarding treatment of *M. marinum* infections. But according to the guidelines of the American Thoracic Society and the Infectious Disease Society of America, a combination of clarithromycin and ethambutol, with the addition of rifampicin for deep structure infection is preferred, and a one drug regimen is discouraged^29^. However, earlier studies have described a successful outcome with a single drug regimen for superficial cutaneous infection^8,10,15^. One-hundred percent of the patients treated with a 3 or 4 drug backbone regime received a combination of rifampicin, clarithromycin and ethambutol, however of the patients receiving a 2 drug backbone regimen only a third received clarithromycin and ethambutol. Notably, the outcome was not significant better in the group receiving clarithromycin and ethambutol. In our study, all patients with type III or type IV infection were treated with a combination of two drugs or more, including clarithromycin and ethambutol. When the study was completed, two of these patients were still on treatment. Both patients had been diagnosed with rheumatoid arthritis and received immunosuppressive therapy and had been treated for *M. marinum* infection for more than 2 years. Furthermore, all patients on immunosuppressive therapy were treated longer than patients who did not receive immunosuppressive therapy. However, the overall outcome was good. In total, 66.6% of these patients were described as improved at treatment termination. This supports earlier studies, suggesting that immunocompromised patients may need a prolonged multi-drug regimen when diagnosed with a *M. marinum* infection^30^.

The strengths of this study are the nationwide design and all cases are culture confirmed. In only 2 cases the patients’ medical records could not be obtained.

Our study has inherent limitations: the true incidence of *M. marinum* infections might be greater than estimated in this study, as we included only patients with culture-confirmed *M. marinum* infections and patients treated based on a clinical diagnosis were left out.

## Conclusion

*M. marinum* infections are rare and there is a long delay from symptom manifestation to diagnosis. The infection is highly related to aquatic exposure, which emphasizes that *M. marinum* should be a differential diagnose in patients with slow-developing cutaneous elements and relevant exposure. Prolonged therapy may be needed in patients receiving immunosuppressive therapy and severe sequelae are rare but often related to deep tissue infection.
